# COLLABORATIVE MENTORING OPPORTUNITIES VIA THE NATIONAL COLLABORATORY TO ADDRESS ELDER MISTREATMENT

**DOI:** 10.1093/geroni/igae098.0268

**Published:** 2024-12-31

**Authors:** Meredith Troutman-Jordan

**Affiliations:** University of North Carolina Charlotte, Charlotte, North Carolina, United States

## Abstract

Elder abuse is a silent public health issue; with nearly 5 million cases of abuse annually, its prevention is a high priority. Nurses and social workers are the largest groups of healthcare professionals to witness, report and advocate for elder abuse. They have both ethical and legal obligations as mandated reporters. However, a majority of elder abuse is not reported. Home health nurses and social workers are in key positions to identify and intervene when elder abuse is suspected. This presentation shares how collaborative mentoring has worked to address elder abuse. In collaborative mentoring, mentees have the opportunity to engage with a mentor and peers, present ideas, exchange knowledge and experience, and be actively engaged. As a NCAEM mentee the author was guided by a mentor in the development and implementation of a focus groups with home health nurses. Pilot data was used to secure funding for an ongoing mixed-methods study. Qualitative findings from phase one include uncertainty, advocacy, and fear, with some overlap in themes from social work and nursing. This data will inform intervention and resource development for home health nurses and social workers. Positive outcomes of this experience were inspiration to serve as a mentor for a subsequent NCAEM mentee, whose project deals with identification and intervention for elder abuse identified within VA homecare services. This presentation will share outcomes from the presenter’s NCAEM project, the process of then serving as a mentor, and highlight opportunities through collaborative mentoring in addressing the issue of elder abuse.

